# Personalized care for complex lives: initial outcomes of a behaviorally-informed complex care intervention

**DOI:** 10.1186/s12913-024-11332-1

**Published:** 2024-08-06

**Authors:** Trygve Dolber, Ryan Muskin, Patrick Runnels

**Affiliations:** 1 Internal Medicine, Case Western Reserve University School of Medicine, Behavioral Health University Hospitals, 11100 Euclid Avenue, Cleveland, OH 44106 USA; 2 Population Health University Hospitals Health System, 11100 Euclid Avenue, Cleveland, OH 44106 USA; 3 Department of Psychiatry, Population Health University Hospitals Health System, Case Western Reserve University School of Medicine, 11100 Euclid Avenue, Cleveland, OH 44106 USA

**Keywords:** Complex, Care, Utilization, Risk, Cost, Healthcare, Personalized, Relationship

## Abstract

**Background:**

5% of patients account for the majority of healthcare spend, but standardized interventions for this complex population struggle to generate return on investment. The aim of this study is the development and proof of concept of an adaptive intervention to reduce cost and risk of readmission for medically high-risk individuals with any behavioral health diagnosis.

**Methods:**

A behaviorally-oriented, personalized care service was delivered using a consultative, team-based approach including a physician, counselor, dietitian and social worker in collaboration with nurse care coordinators. Iterative re-conceptualizations informed tailored treatment approaches to prevent acute decompensation while retraining behaviors that impeded recovery. This service was offered to a small set of members of the employee health plan at University Hospitals Cleveland with an existing behavioral health disorder from November of 2020 to March of 2023. 26 members receiving the service were identified and matched with 26 controls using a risk algorithm. Members and controls were then classified as high utilizers (*n* = 14) or standard utilizers (*n* = 38) based on utilization claims data.

**Results:**

Primary outcomes of this study included medical expenditures (delineated as planned and unplanned spend) and readmission risk scores. Compared to risk-matched controls, both planned and unplanned health care expenditures significantly decreased (*p* < .05) for 7 high utilizers, and unplanned spend only significantly decreased for 19 standard utilizers (*p* < .05). Risk scores, which predict future spend, decreased significantly for standard utilizers (*p* < .05), but not for high utilizers.

**Discussion:**

The value of a behaviorally-oriented personalized care intervention for medically high-risk patients in a commercial insurance population was demonstrated through decreased spend for high utilizers and decreased risk for standard utilizers. Further expansion, refinement, evaluation and scaling are warranted.

## Background

Half of annual healthcare expenditures can be attributed to a small group of patients, many of whom are transient due to effectively treated acute illnesses [1]. However, a persistent subset of members remain indefinitely at high risk of seemingly preventable medical acuity, their needs extending beyond the scope of current healthcare standards [2].

The emerging field of complex care aims to identify and meet those needs. However, interventions have yielded mixed outcomes. For instance, the Camden Coalition garnered national attention by targeting geographic “hot spots” with intensive nursing and case management; however a subsequent randomized controlled trial showed no reduction in readmission rates [2]. Similar findings for other interventions have been attributed to three key factors. First, as above, most patients with high utilization are transient and recover with usual care. Second, persistently high utilizers are heterogeneous and, therefore, poorly amenable to a standardized intervention. Third, costs of care often increase as interventions connect patients to appropriate health care resources [3].

While most interventions have demonstrated reduced utilization for particular subgroups, such as assertively incorporating social and mental health services through case management for patients with lower risk of acuity [4–6], those with persistently high risk have benefitted more from intensive interventions by a multidisciplinary team. These teams typically include a dedicated primary care physician, a dedicated behavioral health practitioner, and eligibility determined following medical chart review [7, 8].

However, existing interventions have primarily focused on federally insured populations, and, therefore, have emphasized addressing social stressors. A significant gap exists in the literature regarding complex care interventions tailored to commercially insured populations, despite their representation among high-cost patients. 5.6% of our ACO commercial members are classified as high risk, and most of these more socially stable patients have a recent behavioral health diagnosis. In this context, interventions may increase efficacy by focusing on behavioral, rather than social, barriers to medical care. This brief report shares the structure, process, and early results of a novel complex care intervention for medically high-risk, commercial managed care plan members with any behavioral health comorbidity that may be impacting care delivery.

## Methods

### Program description

The Personalized Care for Complex Lives (PCCL) program followed best practices, employing a team-based approach, with a psychiatrist, a licensed professional counselor, a dietitian, and a social worker. The program was available to those with high medical risk and any behavioral health diagnosis among the 35,000 individuals covered by the employee plan of a large health system. Two principles novel among complex care interventions included deep integration of behavioral science and a consultative model.

By addressing habitual behaviors (especially those driven by persistent and distressing anxiety or emotional dysregulation) as the primary drivers of treatment resistance, the program builds on past successes that have emphasized the inclusion of dedicated behavioral health specialists. Furthermore, by integrating behavioral health care into primary care, the program aims to improve management of chronic diseases which are often influenced by mental health factors. Depression, which often manifests alongside anxiety, has been called a “pivotal problem” that drives increasing medical complexity and recurrent acuity [9]. Integration of behavioral health care into primary care has improved control of chronic diseases such as diabetes [10]. Within complex care, focusing on patients with a behavioral health diagnosis also serves to decrease heterogeneity. 43% of the 4,936 highest-risk patients had a recent diagnostic code for anxiety, depression, or substance abuse.

While many interventions utilize mental health counselors, the standard approach of counseling is to apply mental health techniques to achieve mental health outcomes. The PCCL program, however, applied the same or similar mental health techniques to target physical health outcomes. Specifically, the program’s therapists are trained to connect unhelpful behaviors to difficulties in engaging with physical health care, a process not explicitly discussed in reports of other complex care programs.

A behaviorally-informed intervention is best delivered through consultation. Through consultation, patients gain insight into their behaviors and the rewards of trust-building, while the consultation team can incorporate relevant health system context. Existing care teams, which are maintained, benefit from repairing a relationship and delivering effective care. Additionally, they can adapt their processes to better serve similar patients. This consultative model already exists in inpatient consult-liaison psychiatry, which incorporates patient-care team dynamics into its conceptualizations and recommendations [11, 12].

These principles formed the basis of the PCCL intervention. Referrals required both a high risk of acute illness and the presence of a mental health diagnosis (suggesting presence of relevant behavioral factors). The risk category was determined by the Health Risk Monitor, a system developed by Health Cost & Risk Management, LLC., which uses a proprietary algorithm that applies stratified regression formulas incorporating claims data, demographics, expenditure, comorbidity, and acute care data to estimate the risk of future health outcomes, utilization, and costs. Additionally, relevant behavioral factors were implied by existing diagnoses, self-reported stress or depression, or any subjective and unexplained failure to achieve medical stability. Most referrals came from intensive nurse case managers, who telephonically engaged with or coordinated care for patients with increasing risk. The team’s decision to accept each referral was made following chart review and discussion with the referring nurse case manager. Acceptance required the possibility that habitual behaviors were impairing the efficacy of standard care and case management.

Flexibility and creativity in patient engagement was emphasized. If a simple referral from the case manager proved insufficient, further attempts were made based on pre-engagement behavioral conceptualizations. Existing care was leveraged, such as by visiting during inpatient care or collaborating with trusted care providers. The program was presented as a personalized tool for the patient’s desired use, not solely to improve physical health. Initial assessment with the team psychiatrist reinforced this approach, began conceptualization and psychoeducation, and produced an agreed-upon starting plan.

The team met weekly to conceptualize and coordinate patient care. Behavioral conceptualization and engagement was based on the principles of Acceptance and Commitment Therapy (ACT), a trans-diagnostic, mindfulness-based therapy that helps identify cherished values, uncover habitual behaviors that inhibit them, and habituate new behaviors that support them [13]. The psychiatrist and counselor received mastery certification in Focused ACT, which is highly flexible in its delivery [14]. The dietitian was trained in Intuitive Eating, a mindfulness-based approach to eating that prioritizes healthy behaviors over weight loss.

Individualized conceptualizations and plans were dynamically adjusted as barriers were overcome, awareness expanded, and problems that had long been avoided or suppressed came back into focus. Individualized criteria for program completion were set on a case-by case basis, requiring a noticeable shift in personal health care behaviors from avoidance- to value-based.

### Program evaluation

The patients included in this study consisted of ACO members who are commercially insured through the hospital system employee health plan and were successfully referred to the program. Patients were only enrolled if they had any mental health code (other than for dementia or intellectual or developmental disability) billed in the previous two years, including substance use disorders, psychotic disorders, mood disorders, anxiety disorders, behavioral syndromes associated with physical factors, personality disorders, and attention deficit disorder. The final sample consisted of all patients who were enrolled in the program for at least six months (26 in total), regardless of whether they were still enrolled (15 enrollees), had dropped out (5 enrollees), or had already completed (6 enrollees) at that time of analysis. The average time of enrollment until completion was 9 months. In terms of demographics, within the intervention group, 27% were male and 73% were female, with 65% identifying as white, 31% as black, and 4% as other, with a mean age of 48.2 years. The majority of mental health comorbidities were anxiety and depressive disorders.

The control group consisted of 26 randomly selected, risk-matched, non-referred plan members. Demographically, this group comprised 31% males and 69% females, with 73% identifying as white, 23% as black, and 4% as other, with a mean age of 40.9 years. To determine the differential impact of high baseline spending, individuals were categorized as “High Utilizers” (HU) if their monthly medical spending was in the upper quartile of the total population (*n* = 14), while those whose spending fell below this threshold were categorized as “Standard Utilizers” (SU) (*n* = 38). The data collected for this study were accessed through medical claims and protected health information (MRN, date of birth and names). PHI was obtained in order to validate all claims and risk data. Date of birth was used to assure all patients were over 18 years of age. To ensure the protection of participants’ PHI, a unique study identifier was assigned to each participant with a key that could only be accessed by necessary study personnel, thereby maintaining confidentiality and adhering to privacy regulations.

Pre-intervention monthly medical spend was averaged over two years prior to the date of enrollment. Post-intervention monthly medical spend was averaged over time since the date of enrollment. Change in monthly medical spend was calculated by subtracting average post-intervention spend from average pre-intervention spend. For the analyses, all spend data excluded program related costs. Risk of acuity during the next year was determined, as above, using third-party claims analysis, and was recorded at the date of enrollment and the date of analysis. Risk categories were ordinal on a scale of 1–5 with 1 representing “Well,” 2 representing “Low risk,” 3 representing “Medium risk,” 4 representing “High risk,” and 5 representing “Catastrophic risk.”

Further analysis divided spend into planned and unplanned. Planned spend initially included scheduled appointments and procedures as well as pharmacological claims, while unplanned spend included only emergency department encounters and inpatient admissions. Highly expensive, medically necessary, long-term medications masked significant changes, so pharmacological spend was removed from final results. One-way ANOVA was conducted separately for high and standard utilizers, comparing intervention and control groups on the change in overall, planned, and unplanned monthly medical spend. No covariates were included due to the absence of significant differences in sample characteristics. A significance level of 0.05 was used for all tests. All analyses were conducted using R version 4.3.0.

## Results

Results revealed a significant difference in overall monthly medical spend for high utilizers, where the intervention group showed a decrease (M =$-1468.23, SD = $379.41) compared to the control group that had a lower decrease (M =$-99.57, SD =$251.10), F(1,12) = 6.08, *p* = .028. However, for standard utilizers, there was no significant difference in monthly medical spend between the intervention (M =$151.70, SD =$90.92) and control groups (M =$113.47, SD =$104.16), F (1, 36) = 1.26, *p* = .85.

**Fig. 1:**
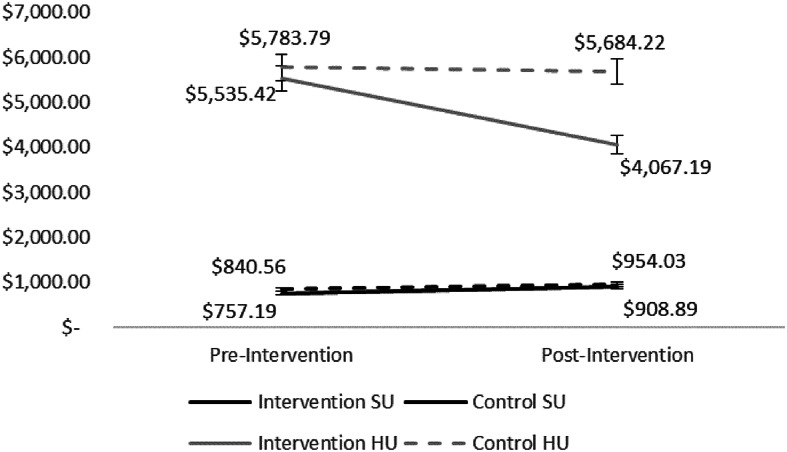
Pre / post avg. monthly medical spend. *Note* Figure depicts average monthly medical spend for both high utilizers and standard utilizers. These were separated out to demonstrate meaningful differences in the effect of the intervention on specific risk and spend groups

There was a significant difference in unplanned spend between intervention and control groups for both high utilizers, F(1,12) = 29.03, *p* < .01, and standard utilizers, F(1,36) = 22.74, *p* < .05. Regardless of utilization level, unplanned spend decreased for the intervention group (HU: M = -$300.95, SD = 88.45; SU: M = -$55.62, SD = $56.39), and increased for the control group (HU: M = $1.42, SD = 71.91; SU: M = $12.32, SD = $43.27).

For planned spend, a significant difference was observed only for HU, F(1,12) = 6.078, *p* = .013, with a larger decrease in the intervention group (M = $-1167.68, SD = $389.90) compared to controls (M = $-100.99, SD = $381.94). There was no significant difference in planned spend for SU, F (1, 36) = 3.281, *p* = .25, as both intervention and control group spend increased (intervention: M = $207.32, SD = $114.81; control: M = $101.15, SD = $92.57).

Change in risk category was evaluated using paired samples t-tests, revealing a significant decrease in risk for the intervention group, from 4.73 (SD = 0.21) to 4.27 (SD = 0.58). Among SU in particular, mean risk decreased from 4.62 (SD = 0.26) to 3.92 (SD = 0.41), a clinically significant change from high risk to medium risk. However, risk did not significantly decrease for HU (baseline M = 4.89, SD = 0.11; post-intervention M = 4.78, SD = 0.44). Risk did not decrease significantly for the control group, regardless of utilization level.

**Fig. 2:**
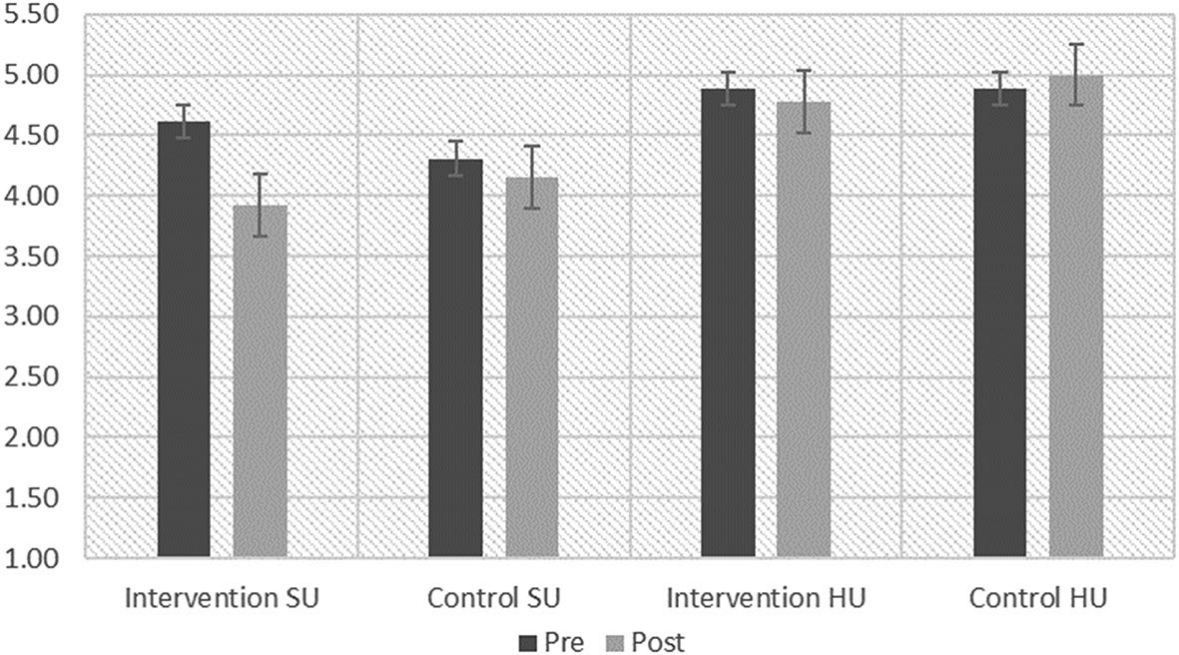
Pre/post population health risk score. *Note* Fig. 2 depicts the pre and post intervention risk scores for both control and intervention groups. Notably, baseline risk scores were not significantly different (*p* = .31) between the treatment and control group

## Discussion

This behaviorally-focused, team-based, multidisciplinary, consult intervention significantly reduced current health care expenditures and risk of medical acuity for patients with commercial insurance, high risk of medical acuity, and any behavioral health diagnoses. Reduction in spend, especially unplanned, was more pronounced for those with higher levels of utilization, and reduction in risk was only significant for those with lower levels of utilization. Because risk of acuity predicts future spend, reduction of risk may be a reliable surrogate for reduction of future spend. This suggests that risk may be useful as an outcome measure when study duration is too short to detect change in spend. Further investigation using a variety of transparent risk algorithms would be necessary to explore this possibility.

Although spending decreased for high utilizers, their risk category remained unchanged. This outcome could be attributed to the intricacies involved in determining risk, which encompass a blend of demographic, social, and clinical factors. While the intervention may effectively curtail medical expenses and unplanned utilization, individuals might remain at high risk due to the persistent or severe nature of their chronic conditions or other criteria unaffected by the intervention. In essence, while financial metrics may show improvement, the underlying risk profile might remain unaltered, highlighting the complexities inherent in addressing chronic health challenges comprehensively.

Standard utilizers, on the other hand, saw a decline in risk without a corresponding reduction in spending. This phenomenon could stem from heightened engagement with healthcare services, particularly among individuals who may have previously underutilized the healthcare system. For instance, risk levels among standard utilizers might have been influenced by factors such as low appointment attendance or low adherence to diagnostic workup, screening measures, and pharmacologic treatment. By addressing these behavioral challenges, it’s reasonable to anticipate a decrease in risk, potentially leading to an increase in spending as patients access necessary care more regularly.

Reduction in spend and risk cannot be assumed to reflect improved health and function. While the intervention team subjectively reported overall improvement, other measures of health, behavior, and function were not collected or analyzed for this brief report. However, reductions in both risk and ratio of unplanned to planned spend are suggestive of increased overall engagement and biopsychosocial stability. Future research of this intervention should include measures of behavior and function as necessary outcomes given the nature of the intervention.

Our analysis revealed that the program incurs annual costs of $375,000 (not including costs saved by billing, i.e., physician and counselor services). However, the intervention group’s cost savings compared to the control group resulted in a projected annual net loss of $162,448. This loss is due to the program cost outweighing the savings of $212,502 generated by the 26 intervention patients. Notably, these savings were primarily driven by the high utilizers. Despite the current loss, the intervention demonstrates potential for significant cost savings when scaled to target a larger population of high utilizers.

Statistical power was limited by sample size and study duration. Despite this, statistically and clinically relevant changes were observed. The risk-matched control group was subject to selection bias, as their inclusion did not require chart review by the clinical team. Some intervention enrollees were simultaneously enrolled in other ACO services, such as focused diabetes or cardio-metabolic management interventions, but random control selection increased the likelihood of equal distribution between groups. Additionally, all enrolled patients were ACO members and enrolled in the employee health plan, thus limiting the generalizability of the results. Future research of this intervention should be applied to a more generalizable sample.

Notably, the results may be limited because variations in primary health and mental health diagnoses were not considered. Future research on this intervention should include physical and behavioral health diagnoses as potential covariates, as different diagnostic groups may be differentially impacted by the intervention. Since no mental health diagnoses were excluded from this study, it is possible that certain conditions, such as substance use disorders, may exhibit different outcomes than others, such as psychotic disorders.

Accounting for these limitations, the results indicate a potential value of the intervention. This may be due to its adaption to the common challenges of transience, heterogeneity, and increasing spend due to higher engagement. Transience was obviated by selection of patients with chronically high risk, rather than acute high utilization. Heterogeneity was reduced by focusing on behavioral health diagnoses, which were highly prevalent. Where higher engagement made decrease in current spend a poor marker of improvement, change in risk category may have been an appropriate surrogate.

## Conclusion

This study found that proven complex care methods can be successfully adapted for a commercial insurance population. The lower burden of social stress shifted the intervention focus to behaviorally-informed complexity, which could also be relevant for populations with higher social stress. This interventions approach can be used to integrate awareness of complex relationships between behavioral, social, and physical stressors to increase conceptual accuracy, identify target processes, and build trust. Future analysis of this intervention will address several limitations. First, the intervention has already expanded to include non-commercial plans, allowing for increased power and generalizability. Second, transparent measures of risk will be applied, providing insight on the validity of risk and its elements as an outcome measure. Third, control group selection will be enhanced by matching according to disease type and severity, behavioral health diagnosis, and social determinants of health. Finally, other relevant outcomes measure will be added, such as quality of life, patient enablement, and disease-specific markers. Together, these will allow for a much more robust analysis, guiding further intervention development and standardization.

## Data Availability

The datasets generated and/or analyzed during the current study are not publicly available due institutional policies regarding patient data safety and monitoring, but are available from the corresponding author on reasonable request.

## References

[1] National Academy of Medicine. In: Finkelman EM, McGinnis JM, McClellan MB, Dzau VJ, editors. Vital Directions for Health & Health Care: an Initiative of the National Academy of Medicine. Washington (DC): National Academies Press (US); 2017. 37782728

[2] Finkelstein A, Zhou A, Taubman S, Doyle J. Health care hotspotting ’ a randomized, controlled trial. N Engl J Med. 2020;382(2):152–62. 10.1056/NEJMsa1906848. 31914242 10.1056/NEJMsa1906848 PMC7046127

[3] Williams BC. Limited effects of Care Management for High Utilizers on Total Healthcare costs. Published online 2015. 26244786

[4] DuBard CA, Jackson CT. Active redesign of a Medicaid Care Management Strategy for Greater Return on Investment: Predicting Impactability. Popul Health Manag. 2018;21(2):102–9. 10.1089/pop.2017.0122. 28968176 10.1089/pop.2017.0122 PMC5906722

[5] Brown DM, Hernandez EA, Levin S, et al. Effect of Social needs Case Management on Hospital Use among Adult Medicaid beneficiaries a Randomized Study. Ann Intern Med. 2022;175(8):1109–17. 10.7326/M22-0074. 35785543 10.7326/M22-0074

[6] Thapa B, Li X, Galárraga O, Impacts of Community-Based Care Program on Health Care Utilization and Cost. 2022;28. Accessed January 27, 2023. https://www.ajmc.com/view/impacts-of-community-based-care-program-on-health-care-utilization-and-cost. 10.37765/ajmc.2022.88862 35420747

[7] Rowe J. Intensive care management of a complex Medicaid population: a randomized evaluation. Am J Manag Care. 2022;28(9):430–5. 10.37765/ajmc.2022.89219. 36121357 10.37765/ajmc.2022.89219

[8] Powers BW, Modarai F, Palakodeti S, et al. Impact of complex care management on spending and utilization for high-need, high-cost Medicaid patients. Am J Manag Care. 2020;26(2):E57–63. 10.37765/ajmc.2020.42402. 32059101 10.37765/ajmc.2020.42402

[9] Safford MM. The complexity of Complex patients. J Gen Intern Med. 2015;30(12):1724–5. 10.1007/s11606-015-3472-6. 26259761 10.1007/s11606-015-3472-6 PMC4636570

[10] Anjana RM, Sagar R, Shankar R, Sridhar GR, Kosuri M, Sosale AR. Effect of a collaborative care model on depressive symptoms and glycated hemoglobin, blood pressure, and serum cholesterol among patients with Depression and Diabetes in India the INDEPENDENT Randomized Clinical Trial. JAMA - J Am Med Assoc. 2020;30322:651–62. 10.1001/jama.2020.11747. 10.1001/jama.2020.11747 PMC7435347 32809002

[11] Tanoubi I, Cruz-Panesso L, Drolet P. The patient, the Physician, or the relationship: who or what is difficult, exactly? An Approach for managing conflicts between patients and Physicians. Int J Environ Res Public Health. 2021;18(23):12517. 10.3390/ijerph182312517. 34886243 10.3390/ijerph182312517 PMC8656806

[12] Groves JE. Taking care of the Hateful patient. N Engl J Med. 1978;298(16):883–7. 10.1056/NEJM197804202981605. 634331 10.1056/NEJM197804202981605

[13] Dindo L, Van Liew JR, Arch JJ. Acceptance and Commitment Therapy: a Transdiagnostic Behavioral Intervention for Mental Health and Medical conditions. Neurotherapeutics. 2017;14(3):546. 10.1007/S13311-017-0521-3. 28271287 10.1007/S13311-017-0521-3 PMC5509623

[14] Linde T, Strosahl K. Doing ACT briefly: the practice of focused acceptance and commitment therapy. In: *Mindfulness and Acceptance in Social Work: evidence-based interventions and emerging applications*. The mindfulness and acceptance practica series. New Harbinger Publications; 2014:163–85.

